# Reported Adverse Events (Side Effects) Following COVID-19 Vaccination Among University Students and Staff: A Case of Islamic University in Uganda

**DOI:** 10.24248/eahrj.v9i1.821

**Published:** 2025-09-30

**Authors:** Rashid Naziru, Madinah Nabukeera Ssebyala, Francis Tamale, Zakariyah Mukasa, John Turyagumanawe, Alex Daama, Areemu Abdul Mujeeb Babatunde, Swaibu Zziwa, Kharim Mwebaza Muluya

**Affiliations:** a Department of Public Health; Faculty of Health sciences; Islamic University in Uganda; b Faculty of Management Studies Islamic University in Uganda; c Ministry of Health Uganda; d Makerere University school of public health; e Rakai health sciences program; f Islamic Medical Association of Uganda (IMAU); g Mayuge Institute for global health sciences research and innovations

## Abstract

**Background:**

Since its outbreak, COVID-19 has brought several disastrous effects on the healthcare and economic systems of different countries globally. There is still no approved curative medicine for COVID-19; thus, the disease can only be controlled through preventive measures, especially vaccination through herd immunity. However, this is still far from being realized, as vaccination is still being affected by hesitancy and resistance from people, mainly due to the fears of side effects and other adverse events following vaccination. Therefore, understanding the incidence and associated factors, nature, and management of these adverse events is paramount for ensuring public confidence in vaccination programmes. The current study therefore aimed at documenting reported adverse events following COVID-19 vaccination among students and staff at the Islamic university in Uganda.

**Methodology:**

This was a cross-sectional study design that was prospective in nature that recruited staff and students from Islamic University in Uganda that received COVID-19 vaccination between February and June 2022. Data was collected using Google Forms; participants were reached through the institutional ERP, emails, text messages and WhatsApp (social media). Data analysis was done using SPSS version 20.

**Results:**

The study recruited 225 participants; 64.0% were female, and the median age range was between 26 and 30 years. Most of the respondents, 76.4%, reported receiving at least one side effect. More than 73% did not report any comorbidity (chronic conditions). 70.5% had received two doses of COVID-19 vaccines, and AstraZeneca was the most received brand at 51.8%. Most participants reported minor side effects, including pain at the injection site (44.4%). fever, chills, headache, and dizziness at 18.1%, and muscle pains and backpains at 14.6%. Only 1.2% reported vaccine sexual reproductive health.

**Conclusions and Recommendation:**

The study confirms that COVID-19 vaccines are safe, as most of the reported side effects were minor with no life-threatening events. More sensitization of the community about the safety of vaccines is encouraged. Ongoing pharmacovigilance surveys for COVID-19 vaccines are recommended to detect possible long-term side effects.

## BACKGROUND

The coronavirus disease 2019 (COVID-19) was declared a global pandemic by the World Health Organization (WHO) on the 11^th^ March 2020. The disease is caused by a novel coronavirus called the severe acute respiratory syndrome coronavirus-2 (SARs-Cov2).^1,2^ Since its outbreak, COVID-19 has brought several disastrous effects on the healthcare and economic systems in the whole world, which greatly impacted the psycho-social, economic, and health aspects of individuals and countries at large.^3,4^ By the end of December 2022, there were over 720 million confirmed cases of COVID-19 and 6.7 million deaths globally. In Uganda alone, there were approximately 169,890 confirmed cases and 3630 deaths according to the WHO.^5^ There is still no approved curative medicine for COVID-19; thus, the disease can only be controlled through preventive measures that include but are not limited to ensuring hand hygiene (frequent hand washing with soap and water, and sanitization using an alcohol-based sanitizer)^6,7^ avoiding crowded places, frequent and regular wearing of face masks,^1,8^ and lastly vaccination.^7,9,10^

Successful vaccination and immunisation remain the main way of controlling and preventing the spread of COVID-19 within communities.^11^ Following the development and the authorisation of the use of several vaccines by the scientific world, vaccination campaigns were launched worldwide to ensure that communities adhered to and responded to the call of being vaccinated. In Uganda, AstraZeneca, Pfizer, Moderna, and Johnson & Johnson, among others, were being used. Uganda, like many other countries, rolled out its COVID-19 vaccination programme in phases, prioritising population groups that were most at risk. These included healthcare workers, the elderly, and individuals with underlying health conditions^12^, and as vaccine availability expanded, the programme extended to include educational institutions, including universities. Vaccination of students and staff at universities was particularly important due to the close-knit and communal nature of campus life, which can facilitate the rapid spread of the virus.^13^ As part of the nationwide vaccination campaigns in Uganda, IUIU initiated a vaccination drive that aimed at safeguarding students, staff, the surrounding communities and Uganda at large.^7^

It should be noted that for vaccination and immunisation to protect the community, a critical mass of the community must be vaccinated and immunised to achieve herd immunity.^14,17^ However, this is still far from being realised, as vaccination is still being affected by hesitancy and resistance from people, mainly due to the fears of side effects and other adverse events following vaccination.^11,18–20^ The situation is even made worse when there are already adverse reactions that have been reported to be associated with COVID-19 vaccination.^21^

Adverse events following immunisation (AEFI) are untoward medical occurrences that follow vaccination and which do not necessarily have a causal relationship with the usage of the vaccine. AEFI encompasses a wide range of reactions that can occur after vaccination, ranging from mild and self-limiting symptoms like pain at the injection site, fever, fatigue, and headache to more serious and rare complications like anaphylaxis or myocarditis.^22^ These events may also be in the form of unfavourable or unintended signs, abnormal laboratory findings, symptoms, or disease.^23,24^. These events should be monitored rigorously by healthcare authorities to ensure vaccine safety and to inform vaccination policies. Several studies have indicated that there are adverse events and side effects reported following COVID-19 vaccination. Furthermore, understanding the incidence and associated factors, nature, and management of these adverse events is paramount for ensuring public confidence in vaccination programmes, which is severely lacking due to the limited information available from the Ugandan population. Given the diverse range of individuals within university populations like IUIU^25^, studying the reported adverse events following COVID-19 vaccination among university students and staff can provide valuable insights into vaccine safety and the unique challenges faced in higher education settings.

Whereas across sectional study done by a pharmacovigilance unit in Portugal found that about 20% of the reported side effects were serious events requiring hospital admissions and some deaths reported^26^, several other studies reported mild to moderate side effects. A systematic review of 11 papers showed that most of the reported side effects were moderate to mild; the same was reported in a facility-based study in Ethiopia by Adisu and others.^26-28^ In all these studies the most reported side effects were pain at the injection site associated with inflammation, redness, fevers and fatigue. ^29^ There are not many studies done in Uganda about reported side effects following COVID-19 vaccination.

The current study therefore documents the reported adverse events following COVID-19 vaccination among students and staff at the Islamic university in Uganda. We specifically looked at the reported side effects, the type of vaccines received, the presence of preexisting health conditions, the time taken to develop the side effects and the duration of healing or disappearance of the side effects. The findings of this study will contribute to the broader understanding of vaccine safety and inform strategies for promoting vaccination campaigns for infectious diseases.

## METHODOLOGY

### Study design

This was a cross-sectional study design carried out on staff and students of the Islamic University in Uganda who received COVID-19 vaccination between the period of February and June 2022.

### Study Population and Sample Size Determination

The study population included students and staff currently enrolled at all the different campuses of Islamic University in Uganda. The university has four campuses, including the main campus in Mbale city, the Arua campus in Arua city, the Kampala campus in Kibuli, Kampala, and the female's campus in Kabojja, Wakiso. Currently, the university has a student population of more than 10,000 students and more than 798 staff.^10,29^ However, this current study focused on students and staff who had received COVID-19 vaccination only.

The following simple formula by Daniel (1999) will be used to determine the sample size^30^



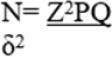



Where N is our sample size.

Z is the standard normal deviation at 95% confidence, equal to 1.96.

*P* is the proportion of the IUIU population that was vaccinated for the COVID-19 vaccine at 20.4%, according to the previous study.^7^

Therefore, our *P* = .204, and Q will be equal to 1-p = 1–0.204 = 0.796.

δ is our maximum acceptable error and was taken as 0.05. Our sample size was 249 participants.

All university students and staff who received a COVID-19 vaccine between February and June 2022 and consented to participate were eligible.

### Study Variables

The dependent variable was the various adverse/side effects reported by the participants. Whereas the independent variables included the social demographic characteristics of participants, the presence of pre-existing medical conditions, the type of vaccines received, the number of doses received, among others.

### Data Collection Methods and Instruments

Information was collected on the individual's gender, age, vaccine dose, and date of vaccination. Information was also collected on specific adverse events, including both local and systemic local and systemic reactions. Local adverse events included injection-site pain, itching, redness, swelling, and induration. Systemic adverse events included fever, headache, generalised body weakness/pain, malaise, muscle ache, joint pain, fatigue, dizziness, chills/fever, vomiting, diarrhoea, abdominal pain, and changed mental status. The severity of adverse events was graded as mild, moderate, and severe according to WHO criteria, as modified by Bae and others ^31^. Outcomes of adverse events were classified into full resolution, resolution with a sequel, and fatality.

Data was collected using Google Forms, which were centrally shared with all staff and students using emails and the university ERP system. (The ERP system is a software system used by organisations to manage and integrate various business processes, including finance, supply chain, human resources, etc.). The instrument used was a self-administered structured questionnaire.

### Data Analysis

Data Analysis was done using the Statistical Package for Social Sciences (SPSS) version 20. Data collected was analysed using descriptive statistics.

### Ethical Consideration and Consent form

Permission to conduct the study within the university was granted by the university secretary through the director of the research, publication and innovation office (RPI). While ethical approval was obtained from the IUIU Research Coordination Committee (RCC) under Ref. Number RCC/FHS/21/007. All participants were made to sign an informed consent (electronically) after fully understanding the details of the study. Participants’ identification was not used anywhere in our research tools, thus ensuring anonymity of our participants. There were no direct benefits or potential risks to the participants related to this study.

## RESULTS

### Sociodemographic Characteristics and Number of Vaccine Doses Received by the Respondents

The majority of respondents (88.9%) were aged 18–35 years, while a small proportion (11.1%) were aged 36 years and older. The results also reveal that the majority of the respondents (64.0%) were females. Most respondents (89.8%) had attained a tertiary or higher level of education. In terms of occupation, the majority of the respondents (44.0%) were medical/nursing students, followed by non-medical students (22.7%). Teachers/lecturers and healthcare workers were 12.0% and 9.8%, respectively. Whereas the majority of the respondents (99.5%) reported receiving the first dose of COVID-19 vaccine, only 70.5% of respondents had received the second dose of COVID-19 vaccine at the time of the study. (Table 1)

**TABLE 1:** Respondents Sociodemographic Characteristics and Number of Vaccine Doses Received

Variable	Respondents in this study (N=225)	
	Frequency	Percentage
Age Group		
18–35 Years	200	88.9
Above 36 Years	25	11.1
Gender		
Female	144	64.0
Male	81	36.0
Education Level		
Post Graduate	54	24.0
Graduate	63	28.0
Tertiary	85	37.8
Secondary	11	4.9
Primary	1	0.4
Others	11	4.9
Occupation		
Student (Medical/Nursing)	99	44.0
Student (non-medical)	51	22.7
Teacher/Lecturer	27	12.0
Health care worker	22	9.8
Lab tech and other office workers 16		7.8
Others	10	4.4
Number of Vaccine Doses received		
One Dose	224	99.5
Two Doses	157	70.5

### Prevalence and Types of Preexisting Medical Condition Before the Vaccination

As shown in Table 2, a large proportion of respondents (73.4%) did not have any pre-existing chronic medical conditions. The remaining had some pre-existing medical condition, with the most common being chronic peptic ulcer disease (10.3%), chronic respiratory illness (4.4%), hypertension (3.6%) and allergies (0.9%).

**TABLE 2:** Prevalence and Types of Pre-existing Medical Conditions Before Vaccination

Variable	Respondents in the Study N=225	
	Frequency	Percentage
Reported Pre-existing Medical Conditions		
No	165	73.4
Yes	60	26.6
Types of Pre-existing Medical Conditions		
Chronic peptic ulcer disease	23	10.3
Chronic respirator-illness (COPD/ASTHIMA)	10	4.4
Hypertension	8	3.6
Pregnant or breastfeeding mother	3	1.4
Diabetes	1	0.4
Hepatitis B	1	0.4
HIV/AIDS	1	0.4
Other chronic illness	9	4.0

### COVID-19 Vaccine Brands Received on the First and Second Doses

As shown in Table 3, out of 224 respondents who reported receiving the first dose of the COVID-19 vaccine, the majority, 51.8%, received AstraZeneca; 36.2% received Pfizer-BioNTech; and 7.1% received Moderna. Only a small proportion of the respondents reported receiving either Sinovac (3.6%) or Johnson & Johnson (1.3%) vaccine brands. Similarly, during the second round of vaccination, the majority of the respondents (33.5%) received the AstraZeneca vaccine, followed by those who received Pfizer-BioNTech (31.2%). A small number of respondents (3.1%) received the Sinovac vaccine brand, and 2.7% received the Moderna vaccine brand. Almost a third (29.5%) of respondents had not yet received their second dose of COVID-19 vaccine by the time of the study.

**TABLE 3:** Vaccine Brand and Determinant of the Brand of Vaccine Received

Variable	Respondents in the Study N=225	
	Frequency	Percentage
Brand of Covid-19 Vaccine Received on the First Dose		
AstraZeneca	116	51.8
Johnson and Johnson	3	1.3
Modema	16	7.1
Pfizer-BioNTech	81	36.2
Sinovac	8	3.6
Brand of Covid-19 Vaccine Received on the Second Dose		
AstraZeneca	75	33.5
Modema	6	2.7
Pfizer-BioNTech	70	31.2
Sinovac	7	3.1
Not received the second dose by the time of study	66	29.5
Determinants for the Brand nCovid-19 Vaccine Received		
It was the only available brand at the facility	176	78.6
The number of doses that are given (one vs. two)	18	8.0
It was recommended to me by a colleague	17	7.6
The nature of side effects	12	5.4
Was recommended by the school	1	0.4

### Determinants of the Brand/Type of Covid-19 Vaccine Received by Respondents.

Respondents outlined the main factors influencing the type of vaccine received (Table 3). These included the availability of the vaccine, with more than 78% indicating this as the reason for receiving the brand they did. Other factors were the number of doses one had to take (8.0%) and recommendation by a friend (7%). A small proportion (5.4%) of the respondents received a particular type of the vaccine because of the nature of side effects.

### Commonest Adverse Events Following COVID-19 Vaccination

The study also assessed the adverse effects reported by the respondents following the first and the second doses of COVID-19 vaccination (Table 4). Of the 224 respondents, 171 (76.4%) reported developing different side effects following their first dose of vaccination, while 22.3% reported no side effects, and less than 2% of the respondents were not sure if they developed any side effects following their first dose of vaccination.

**TABLE 4:** Prevalence of Side effects Following COVID-19 Vaccination and Time to Development of Side Effects

Variable	First Dose		Second Dose^*^	
	Frequency	Percentage	Frequency	Percentage
No	50	22.3	45	28.7
Yes	171	76.4	108	68.8
Don't know	3	1.3	4	2.5
Side Effects Developed After Vaccination				
Pain at the injection site	76	44.4	40	37.0
Fever, Headache, Chills, and Dizziness	31	18.1	19	17.6
Muscle pain and Backache	25	14.6	24	22.2
Cough and flu	16	9.4	16	14.8
Diarrhoea and Vomiting	10	5.8	4	3.7
Difficulties in breathing	8	4.7	2	1.9
Impact on sexuality	4	2.4	3	2.8
Menstrual cycle changes	1	0.6	0	0
Time to Development of the side effects After Vaccination				
Within 6 hours	73	42.7	42	38.9
Within 12 hours	38	22.2	24	22.2
After 24 hours	35	20.5	16	14.8
Immediately	25	14.6	26	24.1

* Only 157 out of 225 reported that they had received a second dose of vaccine

Of the 157 respondents who received the second dose, the majority (68.8%) of the respondents reported having experienced different side effects, while 28.7% reported no side effects. The most reported side effects for both the first and second doses of vaccinations were pain at the site of injection, fever, headache, chills, and dizziness; muscle pain and backache; cough and flu; and diarrhoea and vomiting. Other side effects reported were difficult breathing, erectile dysfunction and menstrual cycle changes.

### Time Between Vaccination and Development of Adverse Events

Table 4 above shows the time taken to develop the different side effects following their first and second doses of vaccination. Of the respondents who developed side effects following their vaccination, most of them experienced side effects within six to ten hours after vaccination, followed by those who reported developing side effects between twelve and twenty-four hours. Whereas in the first vaccination only a small proportion (14%) reported experiencing side effects immediately following COVID-19 vaccination, the proportion of respondents experiencing side effects immediately after vaccination almost doubled (24.1%) in the second dose.

## DISCUSSIONS

The study found that majority of the participants did not have pre-existing medical conditions. This study finding agreed with another study done in Saudi Arabia by Abdul Azizi and others.^32^ This may have been due to the fact that most people with chronic conditions and comorbidities may have been hesitant to receive the COVID-19 vaccinations. The study also found that the majority had received two doses of the vaccines, with the majority receiving AstraZeneca. This finding was also in agreement with the same study done in Saudi Arabia that found that most of the participants received AstraZeneca.^32^ This could have been because AstraZeneca was the most available vaccine in both Africa and the Middle East at the time.

The study further found that the majority of the participants (76.4%) reported at least one side effect. This agreed with most of the studies done elsewhere in different parts of the world.^33,36^ Pain at the injection site, followed by fever, headache, chills, and dizziness, were the commonest reported side effects by the participants. This finding agreed with earlier studies done in Saudi Arabia, Pakistan, Iran, and the Czech Republic that reported minor side effects as the main side effects reported by the participants.^32,36^ This major finding indicates that the COVID-19 vaccine was associated with only minor side effects and no major side effects and not life-threatening side effects.

## CONCLUSIONS

The study found that most of the respondents reported only minor side effects that resolved within a short period of time. No major/life-threatening events were reported by participants. These findings confirm that COVID-19 vaccines were safe and should be encouraged for use.

## RECOMMENDATIONS

There is a need to do more sensitisation and education of the communities towards expected side effects as well as the safety and efficacy of the vaccines. This will increase compliance and minimise or reduce hesitancy of the communities towards the vaccination campaigns. Ongoing vaccine pharmacovigilance surveys for COVID-19 vaccinations should be instituted in order to track long-term side effects.

## References

[1] Rashid N, Nazziwa A, Kantono R, Kasujja H, Zziwa S. Assessing knowledge and practices of the community towards corona virus disease 2019 in Mbale municipality, Uganda: across section study. East African Health Research Journal. 2021 Sep 9;5(1):20–5. 34308241 10.24248/eahrj.v5i1.647 PMC8291215

[2] Rashid N, Nazziwa A, Nanyeenya N, Madinah N, Lwere K. Preparedness, Identification and Care of COVID-19 Cases by Front Line Health Workers in Selected Health Facilities in Mbale District Uganda: A Cross-Sectional Study. East African Health Research Journal. 2021;5(2):144–150. doi: 10.24248/EAHRJ.V5I2.665 35036840 PMC8751497

[3] Haleem A, Javaid M, Vaishya R. Effects of COVID-19 pandemic in daily life. Curr Med Res Pract. 2020;10(2):78. doi: 10.1016/J.CMRP.2020.03.011 32292804 PMC7147210

[4] Pedrosa AL, Bitencourt L, Fróes ACF, et al. Emotional, Behavioral, and Psychological Impact of the COVID-19 Pandemic. Front Psychol. 2020;11. doi: 10.3389/FPSYG.2020.566212 PMC7561666 33117234

[5] World Health Organization. Uganda: WHO Coronavirus Disease (COVID-19) dashboard with vaccination data. 2022 [Internet]. Accessed October 12, 2021. https://covid19.who.int/region/afro/country/ug

[6] Deepa N, Parveen A, Khurshid A, Ramachandran M, Sathiyaraj C, Vimala C. A study on issues and preventive measures taken to control Covid-19. In AIP Conf Proc. 2022;2393(1). doi: 10.1063/5.0075078/2822166

[7] Rashid N, Madinah N, Aisha N, Babatunde AA, Araphat U, Yusuf K. COVID-19 Vaccination: Prevalence and Associated Factors among Students and Staff (A Case of Islamic University in Uganda). Journal of Health Promotion and Behavior. 2022;7(1):18–27. doi: 10.26911/THEJHPB.2021.07.01.03

[8] MacIntyre CR, Chughtai AA, Kunasekaran M, Tawfiq E, Greenhalgh T. The role of masks and respirators in preventing respiratory infections in healthcare and community settings. BMJ. 2025 Feb 27;388. 10.1136/bmj-2023-078573 40015737

[9] Mathieu E, Ritchie H, Ortiz-Ospina E, et al. A global database of COVID-19 vaccinations. Nature Human Behaviour 2021 5:7. 2021;5(7):947–953. doi: 10.1038/s41562-021-01122-8 33972767

[10] Rashid N, Nabukeera M, Kisambira ZJ, Zziwa S, Ndagire MA, Nakayiza F. Knowledge, Attitude and Practices Towards COVID-19 Vaccination Among Students and Staff at the Islamic University in Uganda. EA Health Research Journal. 2023;7(2):339–346. doi: 10.24248/EAHRJ.V7I2.740 PMC12503064 41063773

[11] Daama, A., Rashid, N., Asani, K. et al. Willingness to receive COVID-19 vaccines, associated factors and reasons for not taking a vaccine: a cross-sectional study among persons aged 13–80 years in Wakiso, Central Uganda. BMC Infect Dis 24, 391 (2024). 10.1186/s12879-024-09285-1 38605355 PMC11008005

[12] COVAX roll-out - Uganda. Accessed March 30, 2024. https://www.gavi.org/covax-vaccine-roll-out/uganda

[13] Wilson E, Donovan C V., Campbell M, et al. Multiple COVID-19 Clusters on a University Campus — North Carolina, August 2020. Morbidity and Mortality Weekly Report. 2020;69(39):1416–1418. doi: 10.15585/MMWR.MM6939E3 33001871 PMC7537562

[14] Fontanet A, Cauchemez S. COVID-19 herd immunity: where are we? Nature Reviews Immunology 2020 20:10. 2020;20(10):583–584. doi: 10.1038/s41577-020-00451-5 PMC7480627 32908300

[15] Rashid N, Ddamulira JB, Ndugwa SK, et al. Self-Reported Hepatitis B Vaccination Uptake and Associated Factors Among Adults Attending Budwale Health Center in Mbale District Uganda. EA Health Research Journal. 2023;7(2):347–356. doi: 10.24248/EAHRJ.V7I2.739 PMC11364180 39219647

[16] Rashid N, Swaibu Z. Knowledge, Attitude, and Perception on Hepatitis B Vaccination Among Non-health Workers Attending Selected Health Facilities in Mbale City, Uganda. http://www.sciencepublishinggroup.com. 2021;6(4):139. doi: 10.11648/J.WJPH.20210604.12

[17] Naziru R. Prevalence of Hepatitis B vaccination and associated factors among adults attending Budwale Health Center in Mbale District Uganda (Doctoral dissertation, Makerere University).

[19] Dubé E, MacDonald NE. COVID-19 vaccine hesitancy. Nature Reviews Nephrology 2022 18:7. 2022;18(7):409–410. doi: 10.1038/s41581-022-00571-2 PMC9004449 35414006

[20] Troiano G, Nardi A. Vaccine hesitancy in the era of COVID-19. Public Health. 2021;194:245–251. doi: 10.1016/J.PUHE.2021.02.025 33965796 PMC7931735

[21] Kabagenyi A, Wasswa R, Nannyonga BK, et al. Factors Associated with COVID-19 Vaccine Hesitancy in Uganda: A Population-Based Cross-Sectional Survey. Int J Gen Med. 2022;15:6837–6847. doi: 10.2147/IJGM.S372386 36061966 PMC9432568

[22] Kouhpayeh H, Ansari H. Adverse events following COVID-19 vaccination: A systematic review and meta-analysis. Int Immunopharmacology. 2022;109. doi: 10.1016/J.INTIMP.2022.108906 PMC9148928 35671640

[23] Singh A, Khillan R, Mishra Y, Khurana S. The safety profile of COVID-19 vaccinations in the United States. Am J Infect Control. 2022;50(1):15–19. doi: 10.1016/J.AJIC.2021.10.015 34699960 PMC8539830

[24] Tozzi AE, Asturias EJ, Balakrishnan MR, Halsey NA, Law B, Zuber PLF. Assessment of causality of individual adverse events following immunization (AEFI): a WHO tool for global use. Vaccine. 2013;31(44):5041–5046. Doi: 10.1016/J.VACCINE.2013.08.087 24021304

[25] Bonhoeffer J, Kohl K, Chen R, et al. The Brighton Collaboration: Addressing the need for standardized case definitions of adverse events following immunization (AEFI). Vaccine. 2002;21(3-4):298–302. doi: 10.1016/S0264-410X(02)00449-8 12450705

[26] Rashid N. Sars-Cov-2 B.1.1.529 (Omicron) variant outbreak: case series presentations and response to treatment at the Islamic University in Uganda health facility. ScienceRise. 2022;(1):36–40. doi: 10.21303/2313-8416.2022.002369

[27] Vannacci A, Lombardi N, Crescioli G, Amaro C, Monteiro C, Duarte AP. COVID-19 Vaccines Adverse Reactions Reported to the Pharmacovigilance Unit of Beira Interior in Portugal. Journal of Clinical Medicine 2022, Vol 11, Page 5591. 2022;11(19):5591. doi: 10.3390/JCM11195591 PMC9571682 36233459

[28] Ganesan S, Al Ketbi LMB, Al Kaabi N, et al. Vaccine Side Effects Following COVID-19 Vaccination Among the Residents of the UAE—An Observational Study. Front Public Health. 2022;10. doi: 10.3389/FPUBH.2022.876336/PDFfpubh-11-03604. PMC9120526 35602146

[29] Baden LR, El Sahly HM, Essink B, et al. Efficacy and Safety of the mRNA-1273 SARS-CoV-2 Vaccine. N Engl J Med. 2021;384(5):403–416. doi: 10.1056/NEJMOA2035389 33378609 PMC7787219

[30] Islamic University in Uganda (IUIU). Accessed August 13, 2025. https://www.iuiu.ac.ug/

[31] Bae S, Lee Y, Lim S, Lee J, Lim J, Lee S, et al. Adverse Reactions Following the First Dose of ChAdOx1 nCoV-19 Vaccine and BNT162b2 Vaccine for Healthcare Workers in South Korea. J Korean Med Sci. 2021;36(17):1–9. doi: 10.3346/jkms.2021.36.e115 PMC8093607 33942579

[32] Naing L, Winn TB, Rusli BN. Practical issues in calculating the sample size for prevalence studies. Archives of orofacial Sciences. 2006; 1:9–14.

[33] Alhazmi A, Alamer E, Daws D, et al. Evaluation of Side Effects Associated with COVID-19 Vaccines in Saudi Arabia. Vaccines 2021, Vol 9, Page 674. 2021;9(6):674. doi: 10.3390/VACCINES9060674 PMC8235009 34207394

[34] Abbas S, Abbas B, Amir S, Wajahat M. Evaluation of adverse effects with COVID-19 vaccination in Pakistan. Pak J Med Sci. 2021;37(7):1–6. doi: 10.12669/PJMS.37.7.4522 34912426 PMC8613027

[35] Sabetian G, Moghadami M, Hashemizadeh Fard Haghighi L, et al. COVID-19 infection among healthcare workers: a cross-sectional study in southwest Iran. Virol J. 2021;18(1):1–8. doi: 10.1186/S12985-021-01532-0/FIGURES/2 33731169 PMC7968574

[36] Riad A, Pokorná A, Attia S, Klugarová J, Koščík M, Klugar M. Prevalence of covid-19 vaccine side effects among healthcare workers in the Czech Republic. J Clin Med. 2021;10(7):1428. doi: 10.3390/JCM10071428/S1 33916020 PMC8037149

